# Enyne acetogenins from *Porcelia macrocarpa* displayed anti-*Trypanosoma cruzi* activity and cause a reduction in the intracellular calcium level

**DOI:** 10.1038/s41598-023-37520-3

**Published:** 2023-06-24

**Authors:** Fernanda Thevenard, Ivanildo A. Brito, Thais A. Costa-Silva, Andre G. Tempone, João Henrique G. Lago

**Affiliations:** 1 Centre for Natural and Human Sciences, Federal University of ABC, São Paulo, Brazil; 2 Centre for Parasitology and Mycology, Instituto Adolfo Lutz, São Paulo, Brazil; 3 SENAI Institute of Innovation in Biotechnology, São Paulo, 01130-000 Brazil

**Keywords:** Drug discovery, Plant sciences, Chemistry

## Abstract

Natural products are a promising source of new compounds with a wide spectrum of pharmacological properties, including antiprotozoal activities. Chagas disease, caused by the protozoan parasite *Trypanosoma cruzi*, is one of several neglected tropical diseases with reduced options for treatment, which presents limitations such as toxicity and ineffectiveness in the chronic stage of the disease. Aiming to investigate the Brazilian flora for the discovery of new anti-*T. cruzi* compounds, the MeOH extract from *Porcelia macrocarpa* R.E. Fries (Annonaceae) fruit peels displayed potent activity against trypomastigotes and intracellular amastigotes and was subjected to bioactivity-guided fractionation. Using different chromatographic steps, a fraction composed of a mixture of four new chemically related acetogenins was obtained. The compounds were characterized as (2S*,3R*,4R*)-3-hydroxy-4-methyl-2-(*n*-octadeca-13′,17′-dien-11′-inil)butanolide (**1**), (2S*,3R*,4R*)-3-hydroxy-4-methyl-2-(*n*-eicosa-13′,19′-dien-11′-inil)butanolide (**2**), (2S*,3R*,4R*)-3-hydroxy-4-methyl-2-(*n*-octadec-13′-en-11′-inil)butanolide (**3**), and (2S*,3R*,4R*)-3-hydroxy-4-methyl-2-(*n*-eicosa-13′-en-11′-inil)butanolide (**4**) by NMR analysis and UHPLC/ESI-HRMS data. The mixture of compounds **1–4,** displayed an EC_50_ of 4.9 and 2.5 µg/mL against trypomastigote and amastigote forms of *T. cruzi*, respectively, similar to the standard drug benznidazole (EC_50_ of 4.8 and 1.4 µg/mL). Additionally, the mixture of compounds **1–4** displayed no mammalian toxicity for murine fibroblasts (CC_50_ > 200 µg/mL), resulting in a SI > 40.8 and > 83.3 against trypomastigotes and amastigotes, respectively. Based on these results, the mechanism of action of this bioactive fraction was investigated. After a short-time incubation with the trypomastigotes, no alterations in the cell membrane permeability were observed. However, it was verified a decrease in the intracellular calcium of the parasites, without significant pH variations of the acidocalcisomes. The intracellular damages were followed by an upregulation of the reactive oxygen species and ATP, but no depolarization effects were observed in the mitochondrial membrane potential. These data suggest that the mixture of compounds **1–4** caused an irreversible oxidative stress in the parasites, leading to death. If adequately studied, these acetogenins can open new insights for the discovery of new routes of death in *T. cruzi*.

## Introduction

Chagas disease (CD) is a disease caused by the protozoan parasite *Trypanosoma cruzi*^1,2^. Transmitted through some reduviid hematophagous insects, CD is endemic to South and Central America, with vector-borne and oral as primary forms of transmission in these regions^3^. Due to globalization and human migration, the incidence of this disease increased in urban centers and in non-endemic countries where the principal transmission form is congenital or vertical transmission^4–7^. This disease presents two clinical phases: an acute phase, mostly asymptomatic and with a high blood parasitemia caused by the trypomastigote forms. The chronic phase is characterized by the presence of intracellular amastigotes and 30% of cases can progress to heart/digestive tract mega-syndromes and is the major cause of cardiac diseases in endemic areas^3,8–10^. Infecting more than seven million people worldwide, an estimated seven million are affected in Latin America where being about one million women of reproductive age^1,10^. It is assumed that CD causes about US$ 7 billion/year in production loss, cardiovascular disease treatment, and health care worldwide within US$ 1 billion only in South America^5,6^. Due to a low investment, there are only two drugs available for the clinical treatment, benznidazole and nifurtimox, with efficacy restricted to the acute phase and intense side effects^10,11^. Since cardiac and gastro-intestinal patients are especially susceptive to COVID-19 complications, the outbreak increases the urgency for new drugs and treatments, especially due to impacted health services, and worsening prognostics^12–15^.

In this context, natural products are an alternative in the prospection of new drug prototypes^16^. Previous studies from our research group with *Porcelia macrocarpa* R.E. Fries (Annonaceae) led to the isolation of metabolites with in vitro potential against trypomastigote and amastigote forms of *T. cruzi*. As reported^17,18^, extracts from seeds and flowers afforded acetylene acetogenins and fatty acids with potent activity against trypomastigotes forms of *T. cruzi* caused by depolarization of both the plasma membrane and mitochondrial membrane potential of the parasite. As part of our continuous studies with this plant, the MeOH extract from fruits peels of *P. macrocarpa* was prepared and the effect against trypomastigotes and amastigotes of *T. cruzi* was investigated. As a result, it was observed that this extract caused 100% of parasite death at 300 µg/mL indicating the presence of bioactive metabolites. Using a bioactivity-guided approach, it was obtained a fraction composed of a mixture of four new enyne acetogenins (**1–4**) which were chemically characterized by NMR and UHPLC/ESI-HRMS and evaluated against *T. cruzi* trypomastigotes and amastigotes. Finally, insight into the mechanism of lethal action of the mixture composed by **1–4** was investigated.

## Results and discussion

### Chemical characterization of compounds *1*–*4*

**Figure 1:**
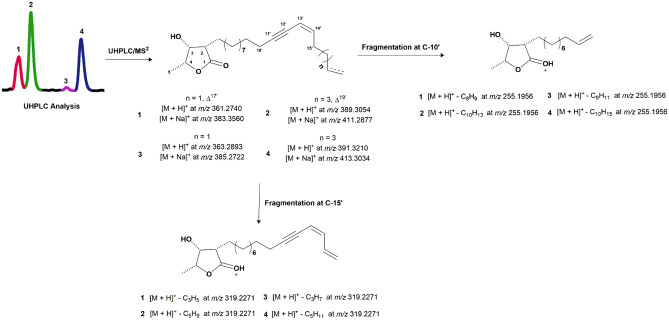
UHPLC-ESI-HRMS/MS (positive mode) analysis of bioactive fraction D-1 from *P. macrocarpa* fruit peels and structures of acetogenins **1**–**4**.

Bioactive fraction D-1 obtained by chromatographic fractionation of MeOH extract from *P. macrocarpa* (see “Methodology” section) fruit peels was analyzed by UHPLC/UV analysis. As could be seen in Fig. 1, this fraction showed to be composed of four related compounds (**1–4**) due to the absorptions at λ_max_ at 212, 267 and 283 nm. According with literature data^19^, this profile is characteristic of enyne conjugated system. Molecular formulas for these compounds were established as C_23_H_36_O_3_ (**1**, [M + H]^+^ and [M + Na]^+^ at *m/z* 361.2740 and 383.2560), C_25_H_40_O_3_, (**2**, [M + H]^+^ and [M + Na]^+^ at *m/z* 389.3054 and 411.2877), C_23_H_38_O_3_, (**3**, [M + H]^+^ and [M + Na]^+^ at *m/z* 363.2893 and 385.2722) and C_25_H_42_O_3,_ (**4**, [M + H]^+^ and [M + Na]^+^ at *m/z* 391.3210 and 413.3034) by ESI-HRMS (Fig. 1). ^1^H NMR spectra of mixture of compounds **1–4** displayed characteristic signals of a γ-lactone ring at δ_H_ 1.43 (d, *J* = 6.5 Hz, H-5), 2.58 (dt, *J* = 4.8 e 3.0 Hz, H-2), 4.31 (dd, *J* = 4.8 e 3.0 Hz, H-3), and 4.46 (dq, *J* = 6.5 e 3.0 Hz, H-4)^18,19^. Signals attributed to hydrogens of a terminal double bond of a alkyl side chain at δ_H_ 5.82 (ddt, *J* = 16.9, 9.9 e 6.8 Hz, H-19′ for **2** and H-17′ for **1**) and 4.97 (m, H-20′ for **2** and H-18′ for **1**) were observed. However, the presence of a triplet at δ_H_ 0.88 (*J* = 6.9 Hz, H-20′ for **4** and H-18′ for **3**), assigned to a terminal methyl group, suggested the presence of related derivatives but containing a sp^3^ carbon at terminal position of side chain, similar of related γ-lactones isolated from seeds of *P. macrocarpa*^20^. Interestingly, ^1^H NMR spectrum showed additional signals at δ_H_ 6.04 (dt, *J* = 10.7 e 7.6 Hz) and δ_H_ 5.48 (t, *J* = 10.7 Hz) attributed, respectively, to hydrogens of an enyne conjugation system, in agreement with data obtained from UV spectrum.^19,20^ ^3^C and DEPT NMR spectra confirmed this proposal, with signals referring to the γ-lactone ring at δ_C_ 177.5 (C-1), 47.6 (C-2), 71.3 (C-3), 78.7 (C-4), and 13.7 (H-5). Signals at δ_C_ 80.1 and 80.2 were attributed, respectively, to sp carbons C-13′ and C-14′ whereas those at δ_C_ 137.6 and 115.2 were assigned to sp^2^ carbons in Z configuration—C-16′ and C-15′, respectively—in order to confirm the presence of enyne system. Furthermore, signals at δ_C_ 139.2 (C-19′ for **2** and C-17′ for **1**) and 114.5 (C-20′ for **4** and C-18′ for **3**), attributed to the terminal double bonds, were also observed. Additionally, these spectra showed a signal at δ_C_ 14.1 (C-20′ for **4** and C-18′ for **3**) confirming the presence of derivatives containing terminal methyl group. The positioning of conjugated triple and double bonds on the alkyl side chain for compounds 1–4 was determined at C-11′ and C-13′, respectively, through MS/MS analysis which exhibited fragments of C-10′/C11′ at *m/z* 255.1957 and of C-15′/C-16′ at *m/z* 319.2270, as showed in Fig. 1. Finally, the relative configuration of γ-lactone ring was determined as *cis,cis,cis* based on values of chemical shifts to carbons C-1, C-2, C-3 and C-4, as observed for chemically related other acetogenins obtained from seeds of *P. macrocarpa*^20^. Therefore, structures of new compounds **1–4** were elucidated as (2S*,3R*,4R*)-3-hydroxy-4-methyl-2-(*n*-octadeca-13′,17′-dien-11′-inil)butanolide (**1**), (2S*,3R*,4R*)-3-hydroxy-4-methyl-2-(*n*-eicosa-13′,19′-dien-11′-inil)butanolide (**2**), (2S*,3R*,4R*)-3-hydroxy-4-methyl-2-(*n*-octadec-13′-en-11′-inil)butanolide (**3**), and (2S*,3R*,4R*)-3-hydroxy-4-methyl-2-(*n*-eicosa-13′-en-11′-inil)butanolide (**4**).

### Anti-*T. cruzi* activity

The mixture of compounds** 1–4** (fraction D-1) displayed EC_50_ values of 4.9 and 2.5 µg/mL against trypomastigotes and intracellular amastigotes, respectively (Table 1), similar to the values obtained for the standard drug, benznidazol (EC_50_ = 4.8 and 1.4 µg/mL, respectively). No cytotoxicity was observed against mammalian NCTC cells to the highest tested concentration (CC_50_ > 200 µg/mL). The mixture of compounds **1–4** showed elevated selectivity indexes for the bloodstream form (SI > 40.8) and for the intracellular form (SI > 83.3) of the parasite. Comparatively, the standard drug benznidazole resulted in SI > 10.8 for trypomastigotes and > 36.4 for amastigotes. Previous studies^18^ evaluated the effect of related acetogenins from seeds of *P. macrocarpa* and compounds with same γ-lactone unity, but containing a side chain with a triple bond at C-11′ and one double bond at C-19′ displayed moderate activity against amastigotes. Otherwise, no effects against *T. cruzi* were observed when a saturated side chain was present, indicating that unsaturation is a crucial feature for the antiparasitic activity. As observed in this work, the effect of the mixture of acetogenins 1–4 against *T. cruzi* was quite similar to the standard drug benznidazole. This data demonstrates that the presence of the enyne conjugated system plays an important role in the potential against the parasite. Therefore, due to the high activity and improvement of SI when compared to benznidazole, the fraction composed by **1–4** was subjected to further biological studies to evaluate its mechanism of action against trypomastigotes of *T. cruzi*.

**Table 1:** Anti-*T. cruzi* activity and cytotoxicity of mixture of acetogenins **1–4** (fraction D-1) from *P. macrocarpa* fruit peels and positive control benznidazol.

Tested material	^a^EC_50_ (µg/mL)		^b^CC_50_ (µg/mL)	^c^SI	
	Trypomastigotes	Amastigotes	NCTC	Trypomastigotes	Amastigotes
Fraction D-1 (mixture of compounds **1–4**)	4.9 ± 0.8	2.5 ± 1.0	> 200	> 40.8	> 83.3
Benznidazol (positive control)	4.8 ± 1.1	1.4 ± 0.6	> 52.0	> 10.8	> 36.4

^a^EC_50_, 50% effective concentration; ^b^CC_50_, 50% cytotoxic concentration; ^c^SI, selectivity index (CC_50_ mammalian cells/EC_50_ parasite form).

### Mechanisms of action studies

The lethal mechanisms of the fraction D-1, containing compounds **1–4**, was performed to evaluate the plasma membrane permeability, the analysis of intracellular calcium levels, the acidocalcisome alkalinization and mitochondrial membrane alterations, including the electric potential, reactive oxygen species and the ATP levels.

#### Plasma membrane permeability

**Figure 2:**
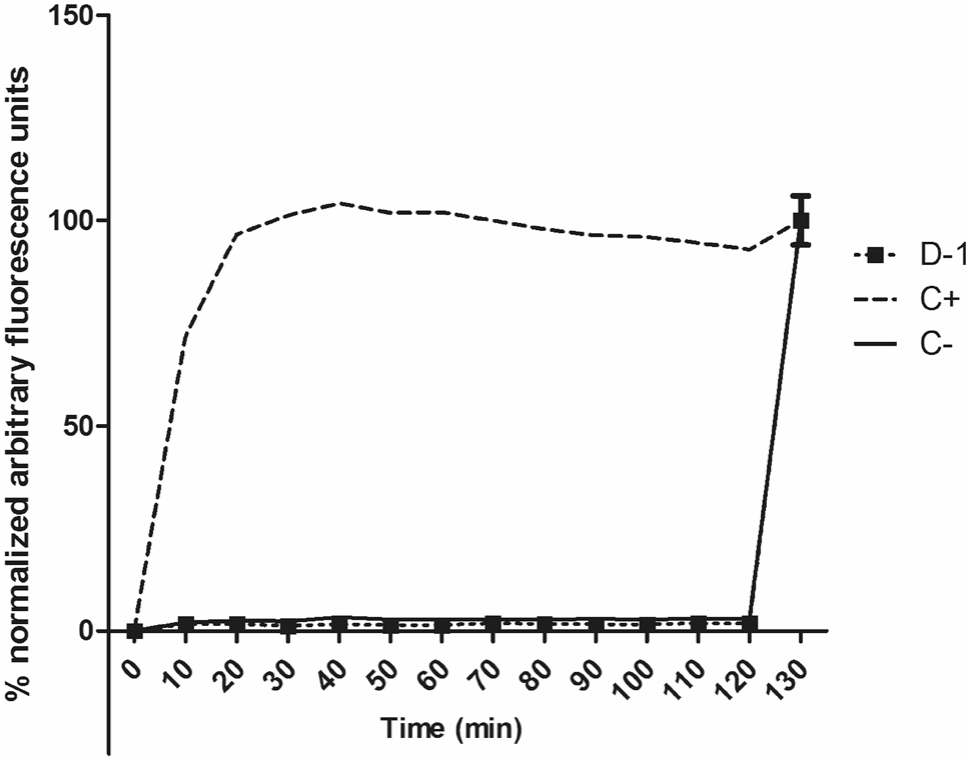
Evaluation of cell membrane permeability effects of the mixture of compounds **1**–**4** (fraction D-1) from *P. macrocarpa* fruit peels at the EC_50_ value of 4.9 µg/mL in *T. cruzi* trypomastigotes. Sytox Green dye (excitation and emission wavelengths of 485 and 520 nm) was used, and fluorescence was monitored until 130 min. Untreated parasites (C−) and Triton X 100 (C+) were used for minimum and maximum permeabilization, respectively. A representative assay is shown.

The plasma membrane regulates transport of ions and nutrients as well as pH and other factors^21^. As such, alterations in its composition can modify the fluidity and contribute to trypomastigote death^22^. Amphotericin B, a clinical drug used in the treatment of *Leishmania* spp., causes permeabilization of the plasma membrane of this protozoan parasite^23^. To evaluate this effect we incubated the mixture of compounds **1–4** (fraction D-1) with trypomastigotes for up to 120 min at the EC_50_ value of 4.9 µg/mL and detected alterations in the fluorescence levels using Sytox Green, a fluorescent probe that binds to nucleic acids and increases the fluorescence after membrane damages^24^. Triton X-100, a non-ionic detergent, was used as a positive control to induce the solubilization of the lipid bilayer^25^. No increases in fluorescence levels were observed after treatment with the mixture of compounds **1–4** (Fig. 2), suggesting no effect in the membrane of the parasite.

#### Intracellular Ca^2+^ levels

**Figure 3:**
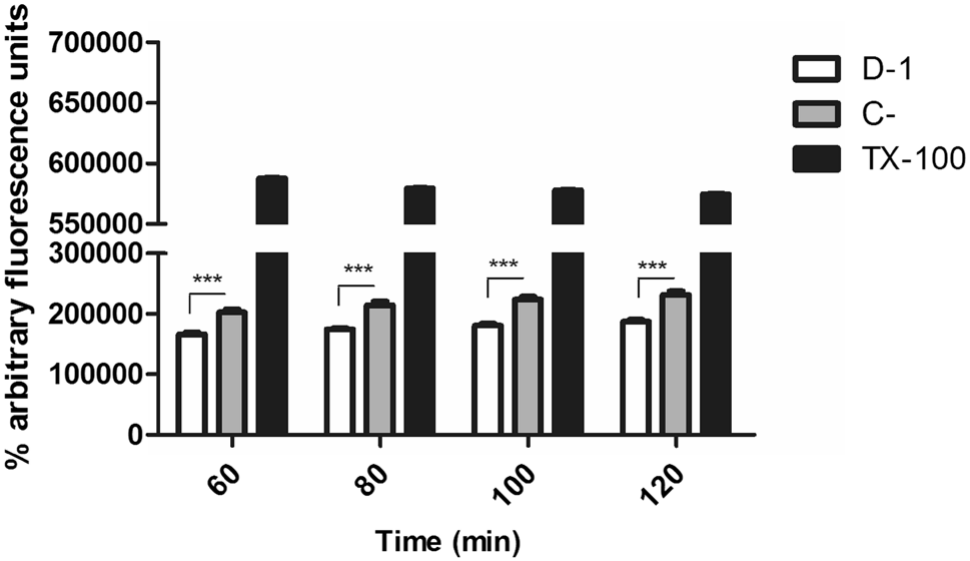
Effect of the mixture of compounds **1**–**4** (fraction D-1) from *P. macrocarpa* fruit peels on intracellular calcium levels in *T. cruzi* trypomastigotes. Using Fluo-4AM dye (excitation and emission wavelengths of 485 nm and 535, respectively), parasites were treated with I for 60, 80, 100 and 120 min at the EC_50_ value of 4.9 µg/mL. Positive (C+) and negative (C−) controls were Triton X-100 (0.5% v/v) and untreated parasites, respectively, Fluorescence in arbitrary units at each time point. A representative assay is shown. ***p < 0.0001.

Calcium ions regulate several signalization pathways, parasite differentiation, flagellar function and are essential for trypanosomatids survival. Alteration of calcium homeostasis can result in the parasite death^26,27^. Using the fluorescent probe Fluo-4AM dye, it was observed that the mixture of compounds **1–4** (fraction D-1) caused reduction of cytosolic Ca^2+^ levels at all evaluated times of the incubation (Fig. 3), suggesting a metabolic imbalance. To evaluate the intracellular calcium, a known organelle in trypanosomatids was studied, named acidocalcisomes.

#### Acidocalcisome alkalinization

**Figure 4:**
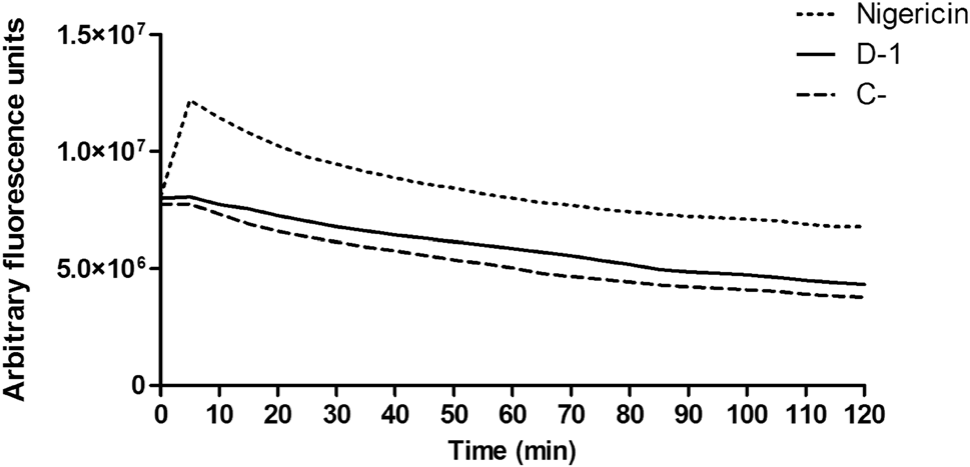
Time dependent analysis of acidocalcisome alkalization of *T. cruzi* trypomastigotes treated with the mixture of compounds **1**–**4** (fraction D-1) from *P. macrocarpa* fruit peels. Pretreatment with acridine orange dye (4 µM) was performed before treating the parasites with the mixture of compounds **1–4** for 120 min at the EC_50_ value of 4.9 µg/mL. Nigericin (4 µM) and non-treated parasites were the positive (C+), and negative (C−) control, respectively. A representative assay is shown.

Acidocalcisomes are acidic organelles and the main Ca^2+^ storage for trypanosomatids, where the ion is bound to polyphosphates^27,28^. The alkalinization of these organelles has been related to leakage of calcium to the cytoplasm^26^. No effect was observed after treatment with the mixture of compounds **1–4** (fraction D-1—Fig. 4), corroborating the reduction of calcium levels in the cytoplasm as previously observed. After treatment with the mixture of compounds **1–4**, the decrease of the calcium levels in the cytoplasmic milieu of the parasite may be a result of the influx of this ion to the mitochondria or the endoplasmic reticulum, since both organelles are natural reservoirs of calcium in the parasite^27,28^. Considering that an elevated concentration of calcium can harm the mitochondria^29^, the electric potential of this vital organelle was further investigated.

#### Mitochondrial membrane potential (Δψm)

**Figure 5:**
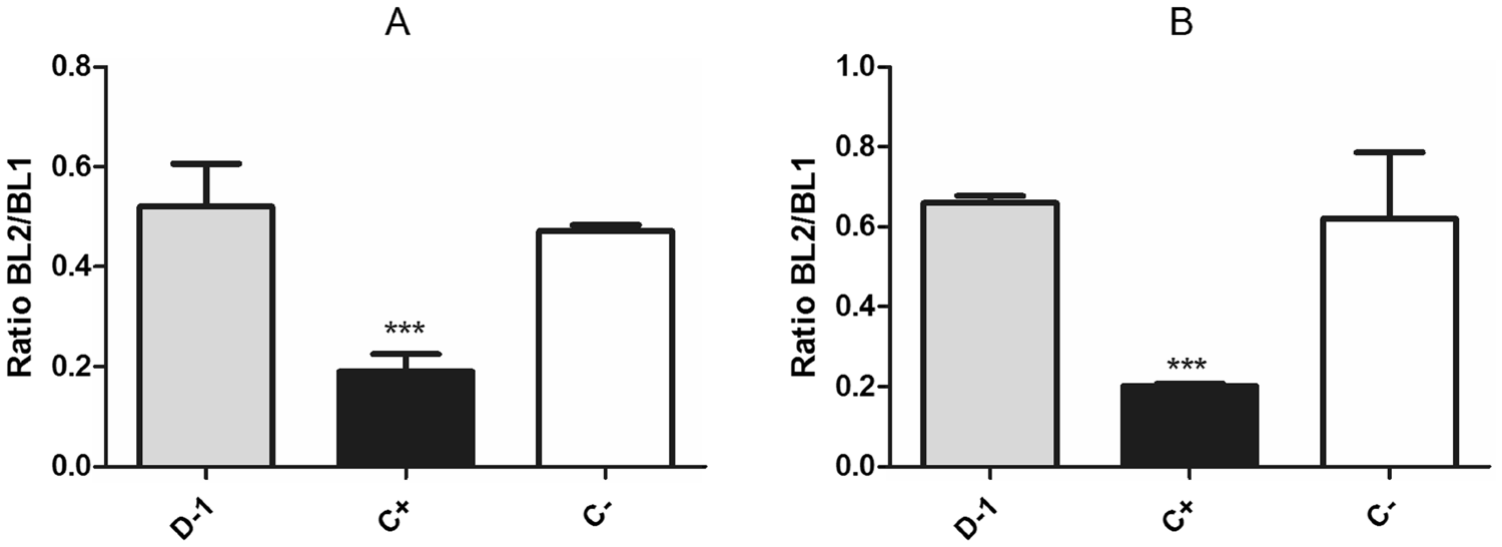
Effect of the mixture of compounds **1**–**4** (fraction D-1) from *P. macrocarpa* fruit peels on the mitochondrial membrane potential of *T. cruzi* trypomastigotes. Parasites were treated with the mixture of compounds **1–4** at 4.9 µg/mL for 1 h (**A**) and 2 h (**B**), using JC-1 dye (excitation and emission wavelengths of 488 nm and 530/574 nm, respectively). Parasites treated with CCCP and untreated parasites were the positive (C+) and negative (C−) controls, respectively. A representative assay is shown. ***p < 0.0002.

The single mitochondrion is unique to the trypanosomatids and is essential for the bioenergetic metabolism. It is also involved in calcium homeostasis as a known source of reactive oxygen species (ROS). Due to these characteristics, the *T. cruzi* mitochondria has been considered an interesting target for new anti-trypanosomal drugs^29,30^. Compounds that affect the respiratory chain tend to impact the ROS production and can also induce oxidative stress. Using the JC-1 probe, alterations in the mitochondrial membrane potential were evaluated. As result, no decreases of the fluorescence levels were detected after treatment with the mixture of compounds **1–4** (fraction D-1—Fig. 5), when compared to untreated parasites. This data reflects no direct impact of the compounds to the organelle. It has been shown that the overloading of calcium by the mitochondria can affect the mitochondrial membrane potential^31^, but this effect was not observed after treatment with the mixture of compounds **1–4**. Although no electric alterations were detected in the mitochondria, alterations of reactive oxygen species levels were studied.

#### Reactive oxygen species levels (ROS)

**Figure 6:**
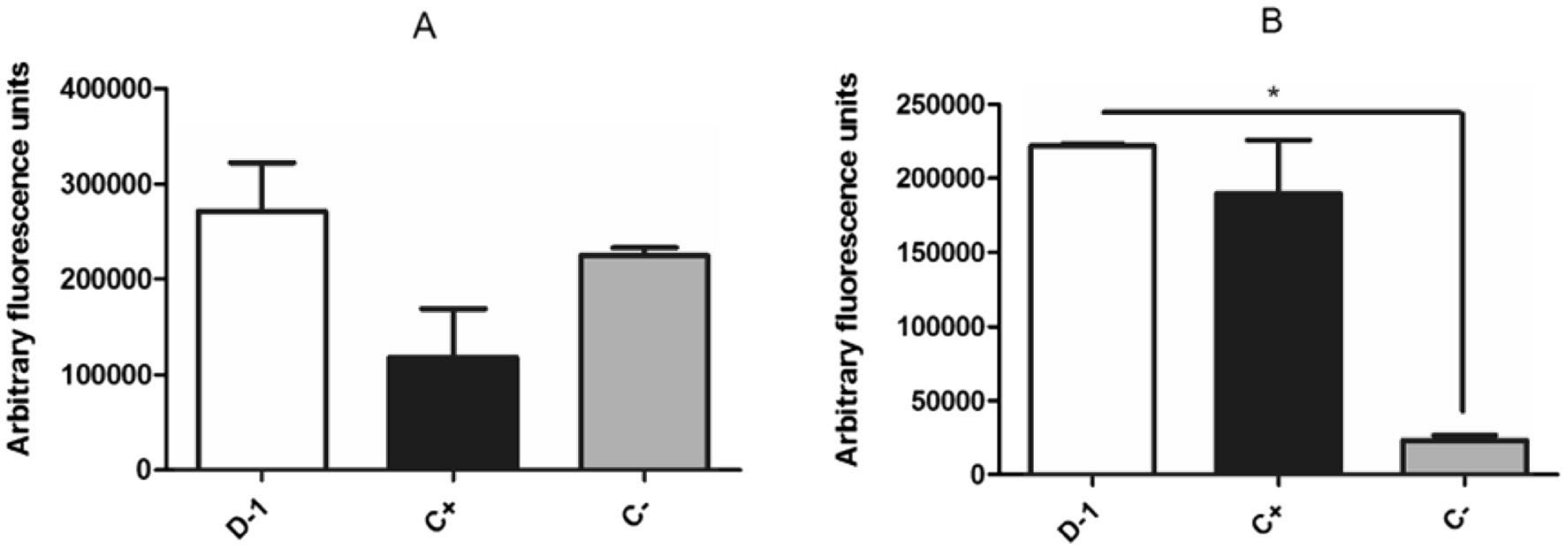
Effect of the mixture of compounds **1**–**4** (fraction D-1) from *P. macrocarpa* fruit peels on the levels of reactive oxygen species in *T. cruzi* trypomastigotes. H2DCFDA dye (excitation and emission wavelengths of 485 nm and 520 nm, respectively) was used in parasites treated with the mixture of compounds **1–4** at the EC_50_ value of 4.9 µg/mL for 1 h (**A**) and 2 h (**B**). Positive control (C+) was 10 mM azide and untreated *T. cruzi* was the negative control (C−).*p < 0.01.

The levels of ROS were also investigated after treatment with the mixture of compounds **1–4** (fraction D-1). Using the fluorescent probe H_2_DCFDA, a significant (*p* < 0.05) increase in ROS levels was observed after 2 h of incubation (Fig. 6). Despite no statistical differences, the ROS levels were clearly increased after 1 h incubation when compared to the untreated parasites. Besides the cellular membrane, endoplasmic reticulum and the cytoplasm, the mitochondria contribute to 90% of the ROS production. The imbalance between mitochondrial reactive oxygen species and removal due to overproduction of ROS and/or decreased antioxidants defense activity results can cause the oxidative stress. These molecules can damage several cellular constituents as DNA, proteins and lipids^32^. Additionally, the detoxification of ROS in Trypanosomatids are done by thiols and the enzyme trypanothione reductase. When these systems fail to cope with an excessive production, these highly reactive molecules (ROS) can induce an oxidative stress, leading to a cellular death^32^. Due to this toxic effect after treatment with the mixture of compounds **1–4**, we investigated the ATP levels.

#### ATP levels

**Figure 7:**
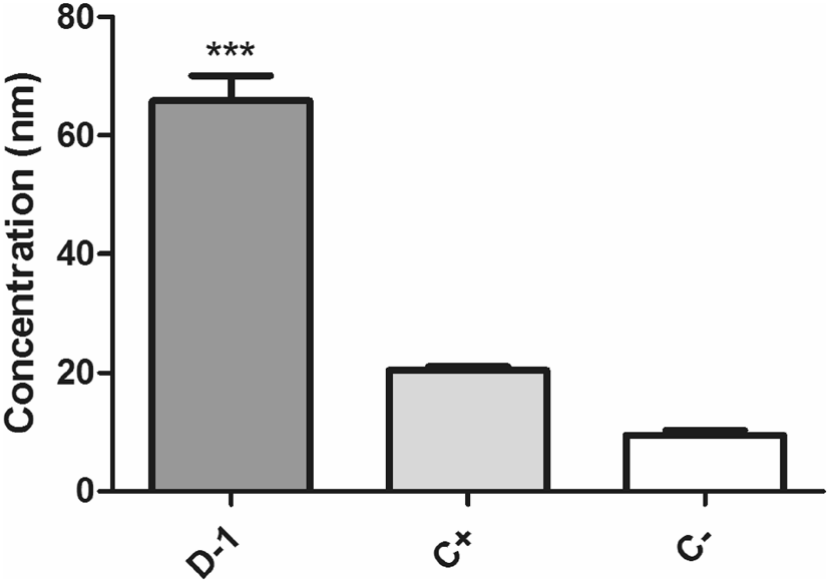
Evaluation of the ATP levels of *T. cruzi* trypomastigotes after treatment with the mixture of compounds **1**–**4** (fraction D-1) from *P. macrocarpa* fruit peels. ATP concentration was measured in a luminometer after 1 h parasite incubation with the mixture of compounds **1–4** at the EC_50_ value of 4.9 µg/mL. Negative (C−) and positive (C+) control were untreated parasites and trypomastigotes treated with 100 µM CCCP, respectively. ***p < 0.0001.

Considering the increased levels of ROS and the possible damages caused by these highly reactive compounds, we investigated the ATP levels of the parasite using a luminescent assay with luciferin. The mixture of compounds **1–4** (fraction D-1) caused a significant increase in ATP levels after 1 h of treatment (Fig. 7) when compared to untreated cells. In parallel, ROS upregulation was also observed after 1 h of incubation as previously discussed (Fig. 6). Increased levels of ROS have been accompanied by elevated release of ATP^33^. Mitochondrial oxidative phosphorylation is the major ATP synthetic pathway in eukaryotes, including Trypanosomatids. During this process, the reduction of O_2_ molecules produces several reactive oxygen species (ROS)^34^. It has also been known that Ca^2+^ is a physiological stimulus for ATP synthesis and consequently, a stimulus for ROS generation^34^. Considering the tight connection among these metabolic compounds, the alteration of any of them can naturally affect the production of the other. In our studies, it is possible that the increased levels of ATP can be a cellular effort to fight the oxidative stress caused by the treatment with the mixture of compounds **1–4**.

## Conclusions

Using a bioactivity-guided approach, four new chemically related acetogenins **1–4** were characterized from the fraction D-1 obtained from the MeOH extract of *P. macrocarpa* fruit peels by NMR and UHPLC/ESI-HRMS analysis. The mixture of compounds **1–4** displayed anti-trypanosomal activity against trypomastigote and amastigote forms of the parasite with a similar effect to the standard drug benznidazole. Additionally, these new compounds showed no cytotoxicity against NCTC cells, demonstrating a promising potential. Due to this activity, mechanism of action studies were performed. The fraction containing compounds **1–4** reduced the cytosolic calcium levels, with no alkalinization of the acidocalcisomes. Although it induced no depolarization of the mitochondrial membrane potential, increased levels of ATP were detected, as a possible cellular effect to counterbalance the oxidative stress caused by the elevated ROS levels leading to the parasite death.

## Methodology

### General experimental procedures

Silica gel 60 PF_254_ (Merck—Darmstadt, Germany) was used for thin layer chromatography (TLC) whereas column chromatography (CC) procedures was performed using silica gel 60 (Merck—Darmstadt, Germany) or Sephadex LH-20 (Sigma-Aldrich–St. Louis, MO, USA). NMR spectra were obtained on a Varian Unity INOVA (500 MHz ^1^H and 125 MHz ^13^C) using CDCl_3_ as solvent and TMS as internal standard. Chemical shifts (δ) are given in ppm with coupling constants (*J*) in Hz. UHPLC experiments were performed on a Thermoscientific Ultimate 3000 model with DAD detector (UVD-170 model) and separation was performed on a Kinetex® C_18_ analytic column (5 µm–250 × 4.6 mm) using ACN:H_2_O 85:15 as eluent at 1.0 mL/min. ESI-HRMS were measured on a Bruker Daltonics MicroTOF QII operating with electrospray ionization (positive mode).

### Plant material

Fruits of *P. macrocarpa* were collected at the *Instituto de Botânica* in São Paulo city, São Paulo State, Brazil (23°38′33.8″S and 46°37′17.5″W) in 2017. Dr. Maria Claudia M. Young identified the plant material, and a voucher specimen was compared with that under number SP76791. This study was registered on the National System for the Management of Genetic Heritage and Associated Traditional Knowledge (SisGen) from the Ministry of the Environment, Brazil, under code A483B45. Experimental research and field studies on plants (either cultivated or wild), including the collection of plant material have complied with relevant institutional, national, and international guidelines and legislation.

### Extraction and bioactivity-guided fractionation

The fruit peels were removed immediately after collection and dried at room temperature. After milling, the plant material (168 g) was defatted (10 × 1L) with *n*-hexane. The residual material was then extracted with MeOH (8 × 1L) at room temperature. After elimination of the solvent under reduced pressure, was obtained the MeOH extract (35 g) which was resuspended in MeOH:H_2_O 1:1 and successively partitioned using *n*-hexane and EtOAc. As both crude MeOH extract and *n*-hexane phase displayed activity against *T. cruzi* (100% of parasite death at 300 µg/mL), part of the *n*-hexane phase (5 g) was fractioned over SiO_2_ column chromatography eluted with increasing amounts of EtOAc in hexane to afford 10 fractions (A–J). Bioactive fraction D (1 g) was chromatographed over Sephadex LH-20 eluted with *n*-hexane:CH_2_Cl_2_ (1:4) and CH_2_Cl_2_:acetone (3:2 and 1:4) to afford eight groups (D-1 to D-8). Fraction D-1 (18 mg) displayed activity against *T. cruzi* and showed to be composed of a mixture of compounds **1–4** by NMR and UHPLC/ESI-HRMS analysis.

### Bioassay procedures

*Animals.* BALB/c mice were utilized for *T. cruzi* maintenance infection and peritoneal macrophage collection^35^. The mice were obtained from the animal breeding facility at the *Instituto Adolfo Lutz*—São Paulo, Brazil and maintained in sterilized boxes with absorbent material under a controlled environment. Food and water were given ad libitum. Procedures were approved by the Ethics Committee of the Instituto Adolfo Lutz (Project CEUA-IAL/Pasteur05/2018), in agreement with the Guide for the Care and Use of Laboratory Animals from the National Academy of Sciences.

#### Parasite and mammalian cell maintenance

Trypomastigotes (Y strain) were maintained in rhesus monkey kidney cells (LLC-MK2—ATCC CCL 7) using RPMI-1640 medium supplemented with 2% bovine fetal serum (BFS) in an incubator humidified with 5% CO_2_ at 37 °C. LLC-MK2 and conjunctive mice cells (NCTC clone L929, ATCC) were maintained at 37 °C with RPMI-1640 supplemented with 10% fetal bovine serum at 5% CO_2_. Macrophages were obtained from the peritoneal cavities of BALB/c mice through washing with the same medium^36,37^.

In vitro activity against *trypomastigote* forms of T. cruzi. Trypomastigotes were obtained from previously infected LLC-MK2 cultures and counted in a Neubauer hemocytometer seeded at 1 × 10^6^ cells/well (96 well plates). The mixture of compounds **1–4** (fraction D-1) was added in serial dilution (100–1.6 µg/mL) using RPMI-1640 medium and the plated were incubated for 24 h at 37 °C in a 5% CO_2_-humidified incubator. Benznidazole was used as the standard drug. Trypomastigote viability was evaluated by the resazurin assay^17,36^.

In vitro activity against amastigote forms of T. cruzi. Previously obtained macrophages were plated on 16 well chamber slides (NUNC®, Merck) at 1 × 10^5^ macrophage/well and incubated for 24 h at 37 °C in a 5% CO_2_-humidified incubator. Trypomastigotes were washed with RPMI-1640 medium and used to infect the cells at 1 × 10^6^/well for 2 h. After this time, non-internalized parasites were removed by washing with RPMI-1640 medium. Serial dilutions (100–1.6 µg/mL) of the mixture of compounds **1–4** (fraction D-1) were incubated with infected macrophages for 48 h at 37 °C and 5% CO_2_ with benznidazole as the standard drug. Slides were then fixed with MeOH and stained with Giemsa. EC_50_ values were obtained after counting infected cells with an light microscope (EVOS M5000) and calculated according to literature^35,37^.

*Cytotoxicity against mammalian cells and selectivity index determination*. Cytotoxicity was evaluated against NCTC cells (L929 clone). Cells were incubated at 6 × 10^4^/well with serial dilutions (200–1.6 µg/mL) of the mixture of compounds **1–4** (fraction D-1) in RPMI medium supplemented with 10% BFS (48 h, 37 °C, 5% CO_2_). CC_50_ values were obtained by MTT assay. Optical density was determined by fluorimetric microplate reader (FilterMax F5, MolecularDevices) at a wavelength of 570 nm^35^. Selectivity indexes (SI) for both forms of T. *cruzi* were calculated using the ratio—SI = CC_50_ against NCTC cells/EC_50_ against parasite forms.

### Mechanism of action studies

*SYTOX Green assay for cell membrane permeability*. Washed trypomastigotes (2 × 10^6^/well) were incubated with 1 µM SYTOX Green probe (Molecular Probes) in HANKS’ balanced salt solution (HBSS, Sigma-Aldrich) supplemented with 10 mM D-Glucose in the dark. The mixture of compounds **1–4** (fraction D-1) was added (t = 0 min) at the EC_50_ value of 4.9 µg/mL and the fluorescence levels were measured every 20 min for 120 min. Untreated parasites were the negative control and maximum permeabilization was achieved with 0.5% Triton X-100. Fluorescence intensity was determined by fluorometric microplate reader (FilterMax F5) with excitation and emission wavelengths of 485 and 520 nm, respectively. Triton X-100 was added to all samples at the end of the assay.

#### Measurement of intracellular calcium (Ca^2+^) levels

Trypomastigotes (2 × 10^6^/well) were pre-treated with Fluo-4AM dye (5 µM) in Phosphate Buffer Solution (PBS) at 37 °C in the dark, for 1 h. Parasites were then briefly washed and treated with the mixture of compounds **1–4** (fraction D-1) at the EC_50_ value of 4.9 µg/mL. Using a fluorometric microplate reader (FilterMax F5 Multi-mode, Molecular Devices), fluorescence levels were measured for 260 min (485 and 535 nm for excitation and emission wavelengths, respectively). Negative control consisted of untreated parasites and maximum intracellular calcium levels were obtained using 0.5% Triton X-100 (v/v)^38,39^.

#### Alterations in acidocalcisomes

Trypomastigotes (2 × 10^6^/well in 96 well plates) were washed with PBS and incubated with acridine orange dye (Thermo Fisher Scientific) at 4 µM for 5 min in the dark. Non-internalized probe was removed with PBS. The mixture of compounds **1–4** (fraction D-1) was incubated with the parasite at the EC_50_ value of 4.9 µg/mL for 120 min. Fluorescence levels were measured using Multimode FilterMax F5 (Molecular Devices) microplate reader with excitation and emission wavelengths of 485 and 535 nm, respectively. Nigericin (4 µM) was used as positive control and non-treated parasites as negative control^39^.

#### Mitochondrial membrane potential (Δψm)

Trypomastigotes (2 × 10^6^/tube) were washed with HBSS supplemented with NaHCO_3_ and D-glucose and incubated with the mixture of compounds **1–4** (fraction D-1) at the EC_50_ value of 4.9 µg/mL, at 37 °C and 5% CO_2_ for 1 and 2 h. After washing, JC-1 dye (10 µM) was added and the parasites were incubated for 20 min at 37 °C in the dark. Fluorescence was measured in a Attune NxTflow cytometer (488 and 530/574 nm excitation and emission wavelengths, respectively). The mitochondrial membrane potential was determined by the ratio between red/green fluorescence intensities (BL-2/BL-1). Positive and negative control were CCCP (Sigma-Aldrich) at 100 µM and non-treated parasites, respectively^39^.

#### Measurement of ROS levels

The mixture of compounds **1–4** (fraction D-1) was incubated at the EC_50_ value of 4.9 µg/mL with washed trypomastigotes (2 × 10^6^/well) in black 96 well plates in HBSS supplemented with NaHCO_3_ (4.2 mM) and D-glucose (10 mM), at EC_50_ value for 1 and 2 h (24 °C, 5% CO_2_). Azide (10 µM) and untreated parasites were used as positive and negative controls, respectively. Afterwards, H_2_DCFDA (20 µM) was incubated with the parasites for 15 min at 37 °C. Fluorescence intensity was measured using FilterMax F5 microplate reader (485 and 520 nm excitation and emission wavelengths, respectively). Controls containing only the compound and the fluorophore were added in all experiments to measure interference^40,41^.

#### Measurement of ATP levels

The mixture of compounds **1–4** (fraction D-1) was incubated at the EC_50_ value of 4.9 µg/mL with trypomastigotes (2 × 10^6^/well) in HBSS and glucose for 1 h, at 37 °C. Parasites were lysed with 0.5% Triton X-100 and mixed with a standard reaction buffer (ATP Determination kit–Thermo Fisher) containing DTT (1 mM), luciferin (0.5 mM) and luciferase (1.25 µg/mL). A luminometer (FilterMax F5 Multi-mode, Molecular Devices) was used for measuring luminescence intensity and ATP quantity calculated with an ATP standard curve^39^.

### Statistical analysis

Samples were tested in duplicates of three independent assays. Calculation of EC_50_ and CC_50_ values was performed using dose–response sigmoid curves in Graph-Pad Prism (GraphPad Prism software 6.0, San Diego, CA, USA). Mechanism of active studies were analyzed using ANOVA turkey test for significance (*p* < 0.05)^38^

## Supplementary Information

Supplementary Information.

## Data Availability

The datasets used and/or analyzed during the current study available from the corresponding author on reasonable request.
