# Supplementary material for: Enyne acetogenins from Porcelia macrocarpa displayed anti-Trypanosoma cruzi activity and cause a reduction in the intracellular calcium level
Source: Sci Rep. 2023 Jun 24;13:10254. doi: 10.1038/s41598-023-37520-3 (PMC10290671; doi:10.1038/s41598-023-37520-3)
Supplement: Supplementary file 1 — Supplementary Information. [file 41598_2023_37520_MOESM1_ESM.docx]

SUPPORTING MATERIAL

Enyne acetogenins from *Porcelia macrocarpa* displayed anti-*Trypanosoma cruzi* activity and cause a reduction in the intracellular calcium level

Fernanda Thevenard^1^, Ivanildo A. Brito^1^, Thais A. Costa-Silva^1,3^,

Andre G. Tempone^2*^, João Henrique G. Lago^1*^

^1^Centre for Natural and Human Sciences, Federal University of ABC, São Paulo, Brazil

^2^Centre for Parasitology and Mycology, Instituto Adolfo Lutz, São Paulo, Brazil

^3^SENAI Institute of Innovation in Biotechnology, 01130-000, São Paulo, Brazil

# *Corresponding authors: [andre.tempone@ial.sp.gov.br](mailto:andre.tempone@ial.sp.gov.br) and [joao.lago@ufabc.edu.br](mailto:joao.lago@ufabc.edu.br)

**Contents**

[**Figure S1** *-* ^1^H NMR spectrum (δ, CDCl_3_, 500 MHz) of fraction D-1 composed of **1 – 4** 3](#_Toc128571795)

[**Figure S2** *-* ^13^C NMR spectrum (δ, CDCl_3_, 125 MHz) of fraction D-1 composed of **1 – 4** 4](#_Toc128571796)

[**Figure S3** - DEPT spectrum (δ, CDCl_3_, 125 MHz) of fraction D-1 composed of **1 – 4** 5](#_Toc128571797)

[**Figure S4** – ESI-HRMS (positive mode) for compound **1** 6](#_Toc128571798)

[**Figure S5** – ESI-HRMS (positive mode) for compound **2** 7](#_Toc128571799)

[**Figure S6** – ESI-HRMS (positive mode) for compound **3** 8](#_Toc128571800)

[**Figure S7** – ESI-HRMS (positive mode) for compound **4** 9](#_Toc128571801)

**Table S1** – ^1^H and ^13^C NMR data to compounds **1** and **3** (δ/ppm, 500 and 125 MHz, CDCl_3_) 10

**Table S2** – ^1^H and ^13^C NMR data to compounds **2** and **4** (δ/ppm, 500 and 125 MHz, CDCl_3_) 11


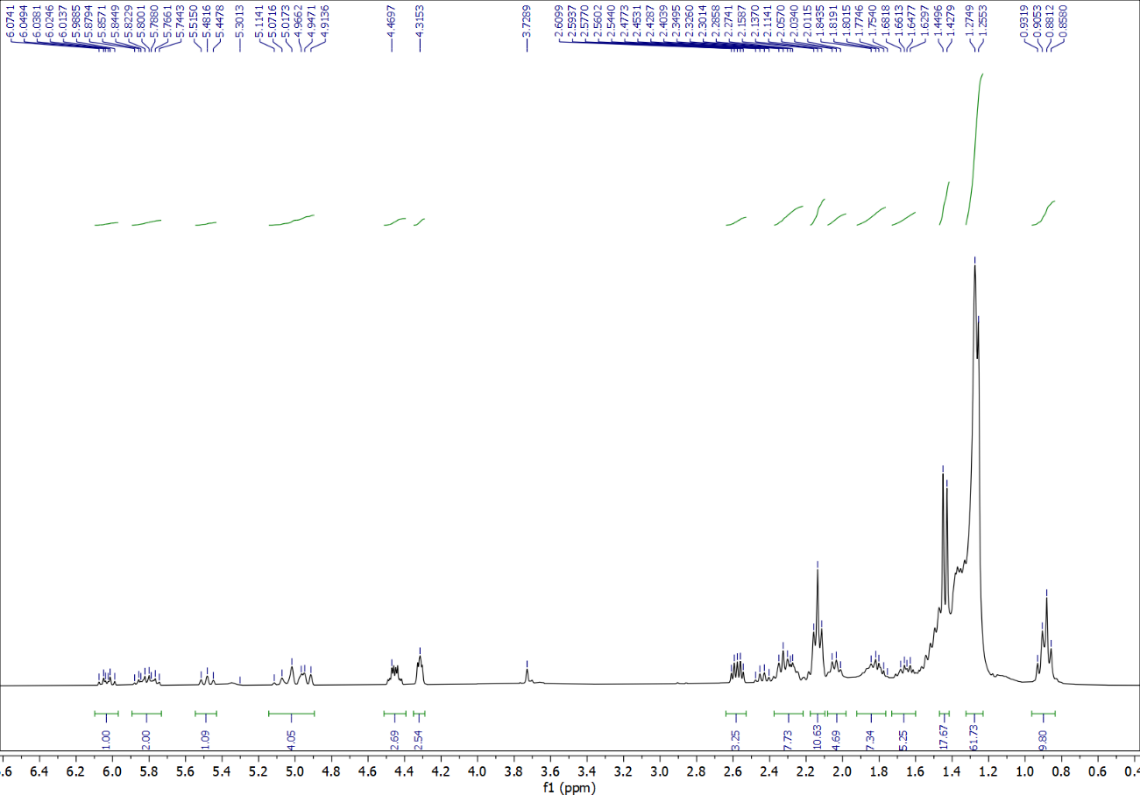


**Figure S1** *-* ^1^H NMR spectrum (δ, CDCl_3_, 500 MHz) of fraction D-1 composed of **1 – 4**


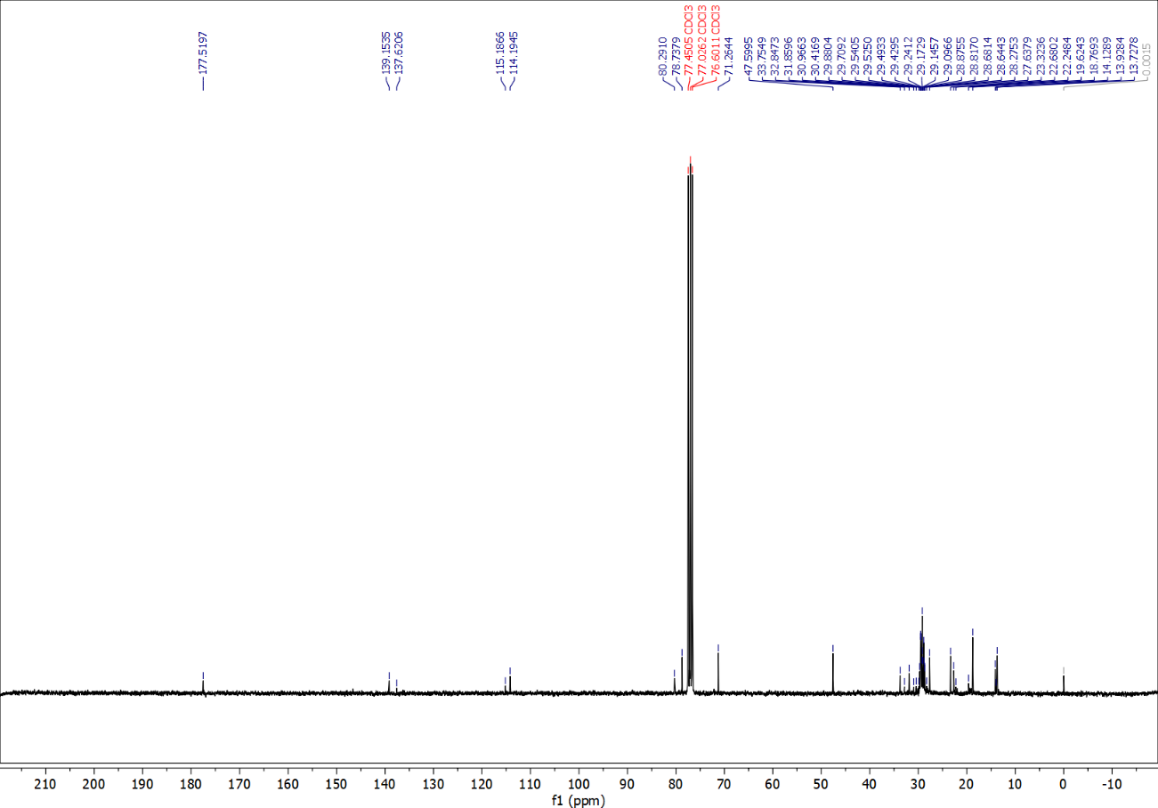


**Figure S2** *-* ^13^C NMR spectrum (δ, CDCl_3_, 125 MHz) of fraction D-1 composed of **1 – 4**


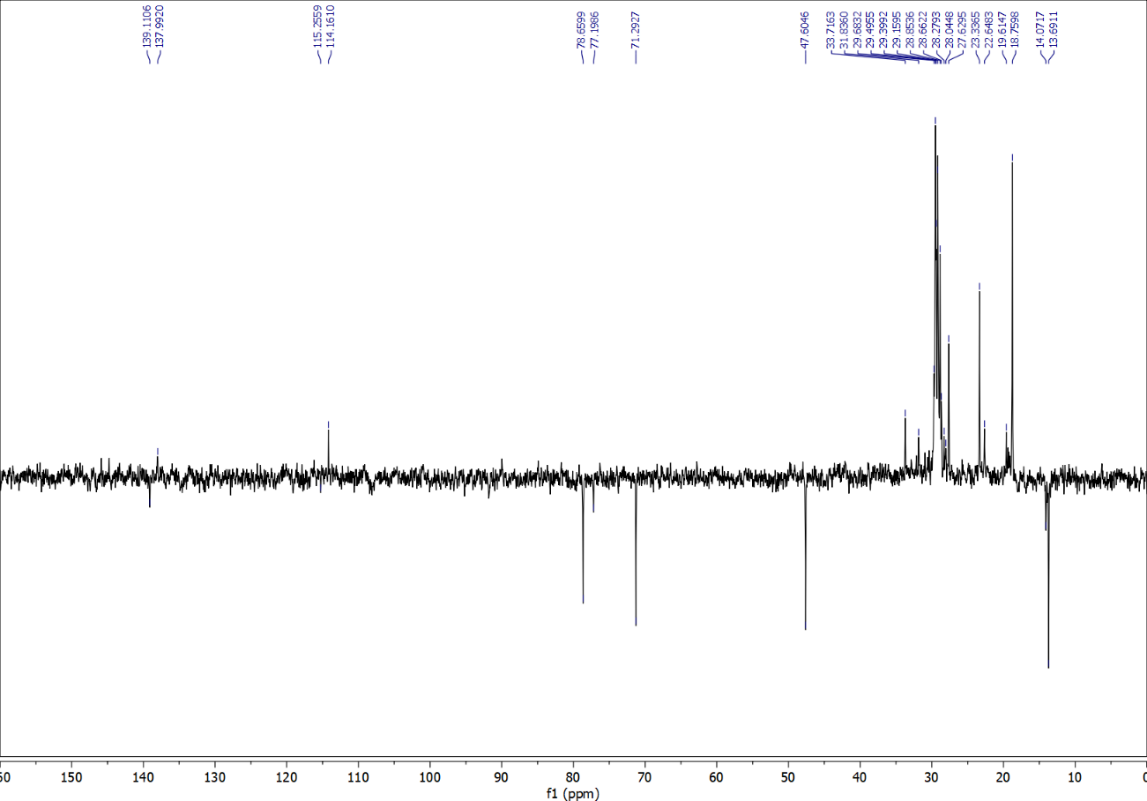


**Figure S3** - DEPT spectrum (δ, CDCl_3_, 125 MHz) of fraction D-1 composed of **1 – 4**


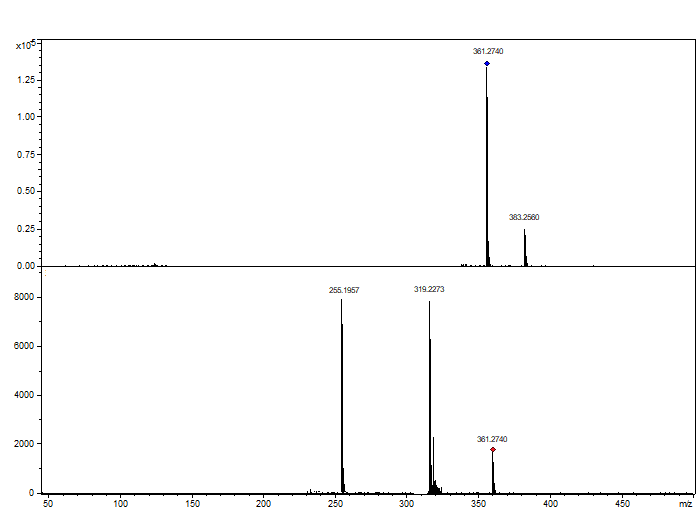


**Figure S4** – ESI-HRMS (positive mode) for compound **1**


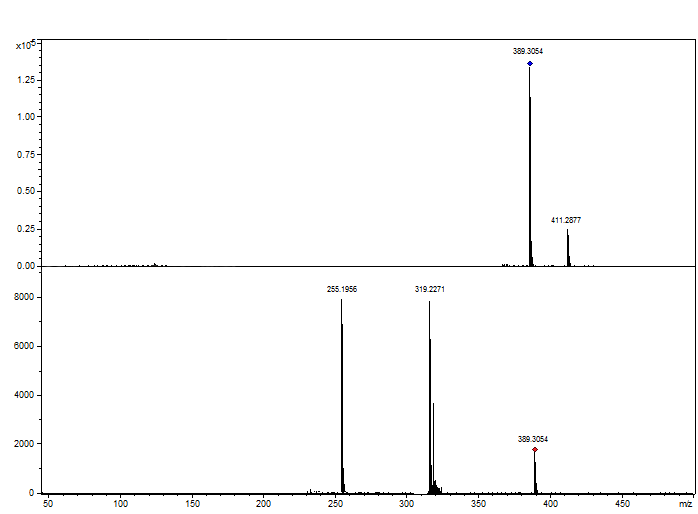


**Figure S5** – ESI-HRMS (positive mode) for compound **2**


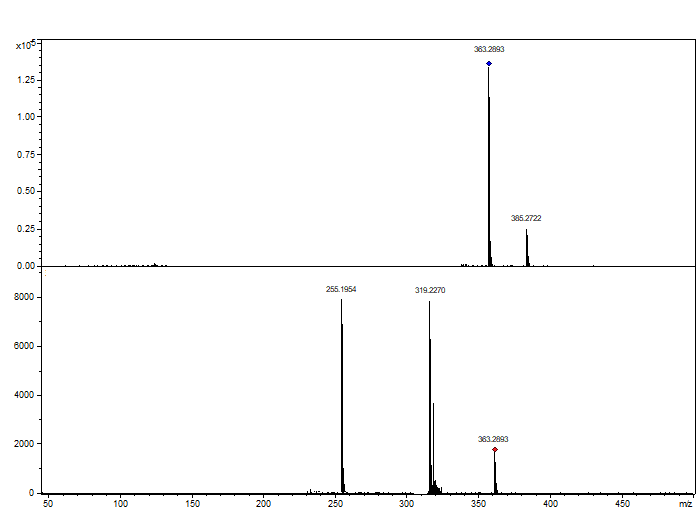


**Figure S6** – ESI-HRMS (positive mode) for compound **3**


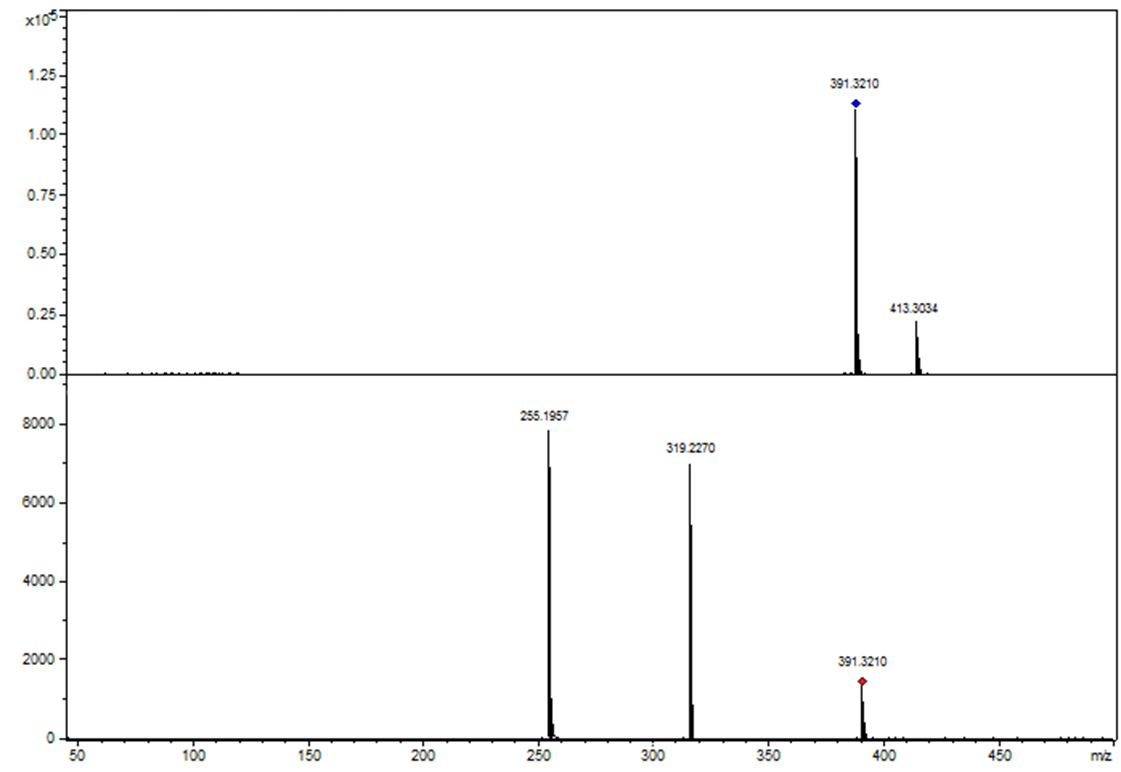


**Figure S7** - ESI-HRMS (positive mode) for compound **4**

**Table S1** – ^1^H and ^13^C NMR data to compounds **1** and **3** (δ/ppm, 500 and 125 MHz, CDCl_3_)

| **position** | **1** | | **3** | |
| --- | --- | --- | --- | --- |
|  | δ_H_ (multiplicity, *J*/Hz) | δ_C_ | δ_H_ (multiplicity, *J*/Hz) | δ_C_ |
| 1 | - | 177.5 (C) | - | 177.5 (C) |
| 2 | 2.58 (dt, 10.0 and 4.8) | 47.6 (CH) | 2.58 (dt, 10.0 and 4.8) | 47.6 (CH) |
| 3 | 4.31 (dd, 4.8 and 3.0) | 71.3 (CH) | 4.31 (dd, 4.8 and 3.0) | 71.3 (CH) |
| 4 | 4.46 (dq, 6.5 and 3.0) | 78.7 (CH) | 4.46 (dq, 6.5 and 3.0) | 78.7 (CH) |
| 5 | 1.43 (d, 6.5) | 13.7 (CH_3_) | 1.43 (d, 6.5) | 13.7 (CH_3_) |
| 1’ | 1.82 (m) | 23.3 (CH_2_) | 1.82 (m) | 23.3 (CH_2_) |
| 2’ | 1.63 (m) | 27.6 (CH_2_) | 1.63 (m) | 27.6 (CH_2_) |
| 3’ - 9’ | 1.27 (br s) | 28.6 – 29.7 (CH_2_) | 1.27 (br s) | 28.6 – 29.7 (CH_2_) |
| 10’ | 2.14 (t, 6.6) | 19.6 (CH_2_) | 2.14 (t, 6.6) | 19.6 (CH_2_) |
| 11’ | - | 80.1 (C) | - | 80.1 (C) |
| 12’ | - | 80.2 (C) | - | 80.2 (C) |
| 13’ | 5.48 (t, 10.7) | 115.2 (CH) | 5.48 (t, 10.7) | 115.2 (CH) |
| 14’ | 6.04 (dt, 10.7 and 7.6) | 137.6 (CH) | 6.04 (dt, 10.7 and 7.6) | 137.6 (CH) |
| 15’ | 1.27 (br s) | 29.7 (CH_2_) | 1.27 (br s) | 29.7 (CH_2_) |
| 16’ | 1.27 (br s) | 33.7 (CH_2_) | 1.27 (br s) | 33.7 (CH_2_) |
| 17’ | 5.82 (ddt, 16.9, 9.9 and 6.8) | 139.2 (CH) | 1.27 (br s) | 22.7 (CH_2_) |
| 18’ | 4.97 (m) | 114.5 (CH_2_) | 0.88 (t, 7.6) | 14.1 (CH_3_) |

**Table S2** – ^1^H and ^13^C NMR data to compounds **2** and **4** (δ/ppm, 500 and 125 MHz, CDCl_3_)

| **position** | **2** | | **4** | |
| --- | --- | --- | --- | --- |
|  | δ_H_ (multiplicity, *J*/Hz) | δ_C_ | δ_H_ (multiplicity, *J*/Hz) | δ_C_ |
| 1 | - | 177.5 (C) | - | 177.5 (C) |
| 2 | 2.58 (dt, 10.0 and 4.8) | 47.6 (CH) | 2.58 (dt, 10.0 and 4.8) | 47.6 (CH) |
| 3 | 4.31 (dd, 4.8 and 3.0) | 71.3 (CH) | 4.31 (dd, 4.8 and 3.0) | 71.3 (CH) |
| 4 | 4.46 (dq, 6.5 and 3.0) | 78.7 (CH) | 4.46 (dq, 6.5 and 3.0) | 78.7 (CH) |
| 5 | 1.43 (d, 6.5) | 13.7 (CH_3_) | 1.43 (d, 6.5) | 13.7 (CH_3_) |
| 1’ | 1.82 (m) | 23.3 (CH_2_) | 1.82 (m) | 23.3 (CH_2_) |
| 2’ | 1.63 (m) | 27.6 (CH_2_) | 1.63 (m) | 27.6 (CH_2_) |
| 3’-9’ | 1.27 (br s) | 28.6 – 29.7 (CH_2_) | 1.27 (br s) | 28.6 – 29.7 (CH_2_) |
| 10’ | 2.14 (t, 6.6) | 19.6 (CH_2_) | 2.14 (t, 6.6) | 19.6 (CH_2_) |
| 11’ | - | 80.1 (C) | - | 80.1 (C) |
| 12’ | - | 80.2 (C) | - | 80.2 (C) |
| 13’ | 5.48 (t, 10.7) | 115.2 (CH) | 5.48 (t, 10.7) | 115.2 (CH) |
| 14’ | 6.04 (dt, 10.7 and 7.6) | 137.6 (CH) | 6.04 (dt, 10.7 and 7.6) | 137.6 (CH) |
| 15’ – 18’ | 1.27 (br s) | 28.6 – 33.7 (CH_2_) | 1.27 (br s) | 28.6 – 33.7 (CH_2_) |
| 19’ | 5.82 (ddt, 16.9, 9.9 and 6.8) | 139.2 (CH) | 1.27 (br s) | 22.7 (CH_2_) |
| 20’ | 4.97 (m) | 114.5 (CH_2_) | 0.88 (t, 7.6) | 14.1 (CH_3_) |
